# Myocardial involvement during the early course of type 2 diabetes mellitus: usefulness of Myocardial Performance Index

**DOI:** 10.1186/1476-7120-6-27

**Published:** 2008-06-05

**Authors:** Paolo Pattoneri, Fabiola B Sozzi, Elisabetta Catellani, Antonella Piazza, Roberto Iotti, Massimo Michelini, Matteo Goldoni, Alberico Borghetti, Maria Domenica Cappellini, Valeria Manicardi

**Affiliations:** 1 Department of Internal Medicine, Nephrology and Health Sciences, University of Parma, Italy; 2 IRCCS Cardiology, Fondazione Ospedale Maggiore, Milan, Italy; 3 Operative Unit of Internal Medicine, "E. Franchini" Hospital, Montecchio Emilia, Italy; 4 IRCCS Policlinico Foundation, Department of Internal Medicine, University of Milan, Milan, Italy

## Abstract

To evaluate whether myocardial performance index detects a subclinical impairment of left ventricular systolic and diastolic function in patients with early stage of type 2 diabetes, without coronary artery disease, with or without hypertension. Furthermore, to evaluate whether some echocardiographic parameters relate to the metabolic control. Fourty-five consecutive male patients (mean age 52.5 years) with type 2 diabetes mellitus of recent onset (23 hypertensives and 22 normotensives) and 22 age matched healthy controls males were analysed. All participants had normal exercise ECG. All subjects underwent standard and Doppler echocardiography for the assessment of the isovolumic Doppler time interval and Doppler-derived myocardial performance index. In all diabetic patients a glycated haemoglobin test was also performed.

No differences were observed in blood pressure, heart rate, and conventional echocardiographic parameters comparing the 2 subgroups of diabetic patients and the controls. Myocardial performance index was significantly higher in diabetic patients independently of the hypertension occurrence, compared to the controls (0.49 and 0.49 diabetic normotensives and hypertensives respectively vs. 0.39, p < 0.01). Myocardial performance index correlated to glycated haemoglobin significantly (r = 0.37, p < 0.01) in both diabetic subgroups. Thus, an early involvement of left ventricular performance was shown by myocardial performance index in patients with type 2 diabetes of recent onset without coronary artery disease, independently of the hypertension presence. These abnormalities can provide a feasible approach to detect a pre-clinical diabetic cardiomyopathy and could be useful for an indirect assessment of the metabolic control.

## Background

A primary diabetic cardiomyopathy represents a high risk factor of heart failure in the absence of ischemic, valvular and hypertensive heart disease in the diabetic population [1-3]. Diabetic cardiomyopathy is characterized by an early left ventricular (LV) diastolic dysfunction and a late LV systolic dysfunction. At conventional echocardiography LV diastolic dysfunction has been documented also in subjects with impaired glucose tolerance [4] and a short duration of type 2 diabetes mellitus [5]. Unquestionably, an early detection of LV damage is a major goal for the prevention of cardiac disease in the diabetic population. Tei et al [6] described a simple, reproducible Doppler derived Myocardial Performance Index (MPI) able to reflect both LV systolic and diastolic function. MPI is diagnostic in patients with heart failure [7,8] and in individuals with overt cardiac disease who have positive risk factors for coronary artery disease [9,10]. Furthermore, it has a prognostic value in patients with myocardial infarction [11], and in those with cardiac amyloidosis [12]. Little is known about the MPI impact in patients with diabetes mellitus of recent onset and without coronary artery disease. The aim of this study was to evaluate whether MPI is able to detect a subclinical LV involvement in patients with a short duration of type 2 diabetes mellitus without coronary artery disease, with and without hypertension, and whether echocardiographic functional parameters, in particular MPI, are related to metabolic abnormalities.

## Methods

### Subjects

The study population consisted of 45 consecutive male patients with type 2 diabetes mellitus of recent onset (23 hypertensives and 22 normotensives) and 22 healthy controls males. The patient population was recruited from the Operative Unit of Internal Medicine, Montecchio Emilia Hospital, IT between July 2006 and December 2006. All partecipants had normal exercise ECG and none was a smoker. All subjects underwent standard and Doppler echocardiography and all diabetic patients also a glycated haemoglobin (HbA_1c_) test. Diabetes mellitus was diagnosed according to the criteria of the American Diabetes Association [13], including fasting plasma glucose level ≥ 126 mg/dl on at least two occasions. HbA_1c _was determined by the HPLC method. Blood sample for analysis was obtained after 8-hours minimum overnight fast and before oral antidiabetic drugs. Hypertension was defined as recommended from the Seventh Report of the Joint National Committee on Prevention, Detection, Evaluation, and Treatment of High Blood Pressure [14] or treatment with antihypertensive drugs for a known diagnosis of hypertension. Body mass index (BMI) was calculated as weight in kilograms divided by the square of height in meters. Obese where those patients who had a BMI > 30 Kg/m^2^. Patients with history of myocardial infarction, chronic stable angina pectoris, previous coronary revascularization, regional wall-motion abnormalities in rest echocardiography, significant valvular disease at echocardiography, significant rest ECG abnormalities suggestive of myocardial ischemia, symptoms or ECG signs of ischemia on exercise stress test, arrhythmias, congestive heart failure, and chronic obstructive pulmonary disease were excluded from the study. The local medical Ethic Committee approved the study protocol. Written informed consent was obtained from all patients.

### Echocardiography

**Figure 1:**
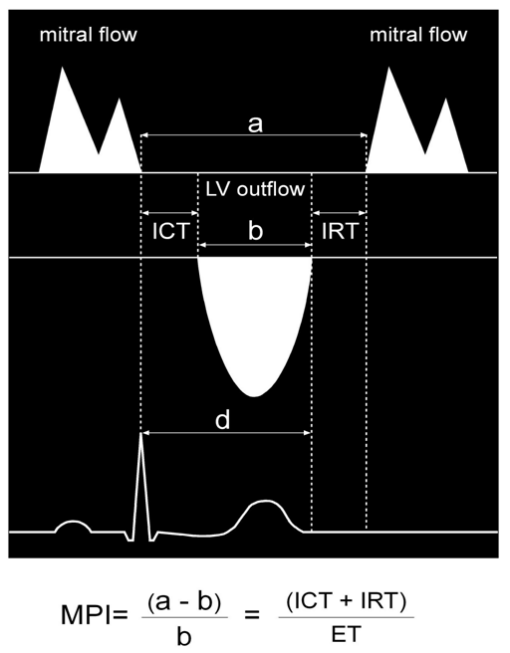
**Flow Doppler at the mitral valve and at the LV outflow tract.** Measurements of the time intervals of the Myocardial Performance Index (MPI). The interval "a" is measured from the end to the onset of mitral inflow waveform; the interval "b" is the left ventricular outflow velocity tracing (ET). The MPI was calculated as (a - b)/b.

All subjects underwent two-dimensional and Doppler echocardiogram according to the recommendations of the American Society of Echocardiography [15]. Recordings were taken from patients standard left lateral decubitus position in expiratory apnoea or quiet breathing by using a multi-hertz sector probe (2–4 MHz) of a Sequoia 512 (Siemens Acuson, Mountain View, California) and stored in the computer of the ultrasonic unit. LV dimensions were measured with M-mode using a leading edge-to-edge convention. The measurements included intraventricular septal thickness (IVS), posterior wall thickness (PW), left ventricular diameter at the end of diastole (LVEDD) and systole (LVESD) respectively. Left ventricular relative wall thickness was calculated as (IVS + PW)/LVEDD. Left ventricular mass (LVM) was calculated using the Penn-convention and indexed to Body Surface Area (LVM/BSA) [16,17]. LV end-diastolic and end-systolic volume as well as the ejection fraction (EF) were calculated according to the biplane Simpson rule. Fractional shortening (FS) was assessed as a percent ratio: (LVDD-LVDS)/LVDD. LV filling was assessed with pulse-wave Doppler echocardiography. Measurements were obtained with the transducer in the apical 4-chamber view, and with the Doppler beam aligned as perpendicular as possible to the plane of the mitral annulus. To obtain mitral flow velocities, Doppler sample volume was placed between the tips of mitral leaflets during diastole. From pulsed Doppler the mitral inflow E-wave deceleration time (DT), peak velocity (pv) and time velocity integral (tvi) of E and A wave and E/A ratios were assessed. These measurements were detected during the second phase of Valsalva manoeuvre in order to discriminate subjects with normal diastolic function from subject with "pseudonormalized" pattern [18]. Overall LV function was assessed using the Tei index, defined as the sum of isovolumetric relaxation and contraction times (IRT and ICT respectively) divided by ejection time [19] which can be obtained from pulsed Doppler recordings of mitral inflow. LV outflow was recorded from the apical long-axis view with pulsed-wave Doppler with the sample volume positioned just below the aortic valve (Figure 1). Other aspects of this technique have been considered in our previous studies [20,21]. All of the echocardiographic measurements were performed by the same experienced cardiologist (P. P.) who was unaware of the patients' clinical data.

### Statistics

All statistical analyses were carried out with SPSS for Windows release 15.0 (SPSS Inc., Chicago, IL, USA). Data are expressed as means + standard deviation. Comparison among the 3 groups for various parameters was performed by 1-way analysis of variance (ANOVA) and the post-hoc Tukey test for multiple comparisons, and two tailed Student's t test for paired data were applied to evaluate differences between controls and patient groups. The relationship between parameters were evaluated by means of simple linear regression data analysis. The *P*-values below 0.05 was considered statistically significant.

## Results

### Baseline Characteristics

The clinical characteristics of the studied population are shown in Table 1. The two groups of diabetic patients were similar for age, diabetes mellitus duration, type of antidiabetic treatment, HbA1c, fasting blood glucose, blood pressure and heart rate at the time of the medical check. Blood pressure and heart rate were lower in the control group.

**Table 1:** Clinical and biochemical characteristics of controls and diabetic patients.

***Parameters***	***Diabetes mellitus (n. 22)***	***Diabetes mellitus and hypertension (n. 23)***	***Controls (n. 22)***
Age (years)	52 ± 7	53 ± 8	53 ± 5
Obesity (%)	18	21	0
Hypertension (%)	0	100	0
Hypercholesterolemia (%)	14	22	0**
Smoking (%)	0	0	0
Family history of myocardial ischemia (%)	15	17	8^†^
Systolic blood pressure (mmHg)	127 ± 5^§^	138 ± 14**	120 ± 14
Diastolic blood pressure (mmHg)	81 ± 7^§§^	82 ± 9**	75 ± 7
Heart rate (bpm)	75 ± 7**	76 ± 10**	67 ± 8
Mean diabetes duration (months)	40 ± 18	36 ± 15	-
Fasting glucose (mg/dL)	167 ± 36^§§^	138 ± 25	87 ± 8
HbA_1c _(%)	7.7 ± 2.2	7.0 ± 1.0	3.7 ± 2.2
Oral hypoglycaemic agents (%)	77	74	0
Diet therapy (%)	23	26	0
Anti-hypertensive theraphy (%)			
ACE-inhibitor	9	65	-
Angiotensin receptor antagonist	-	22	-
Diuretics	-	22	-
Calcium channel blockers	-	22	-
Beta-blockers	-	17	-

**p < 0.01 diabetics vs controls; ^§^p < 0.02 diabetic-normotensive patients vs diabetic-hypertensive patients; ^§§^p < 0.03 diabetic-normotensive patients vs diabetic-hypertensive patients. HbA_1c_, glycated haemoglobin.

### Standard Echocardiographic Parameters

The echocardiographic characteristics of the three study groups are listed in Table 2. LV diameters, volumes, EF, fractional shortening and wall thicknesses were superimposable comparing diabetic normotensive to diabetic hypertensive patients. Diabetic patients showed higher LV diameter, LV volumes, wall thicknesses and LV mass. On the contrary, they showed lower fractional shortening and EF (Table 2). These differences were more clear when diabetes was associated to hypertension.

**Table 2:** Conventional echocardiographic variables in patients and controls.

***Parameters***	***Diabetes mellitus***	***Diabetes mellitus with hypertension***	***Controls***
LV end-diastolic diameter (mm)	50 ± 5	51 ± 5**	46 ± 5
LV end-systolic diameter (mm)	31 ± 5	33 ± 5**	28 ± 4
Septal wall thickness (mm)	10 ± 1^†^	11.1 ± 1**	9 ± 1
Posterior wall thickness (mm)	10 ± 1**	10 ± 1**	9 ± 2
Relative wall thickness	0.42 ± 0.06	0.43 ± 0.06	0.40 ± 0.07
LV mass index (g/m^2^)	116 ± 25**	130 ± 22**	92 ± 23
LV fractional shortening (%)	36 ± 8	35 ± 6	39 ± 5
Ejection fraction (%)	64 ± 6	63 ± 6	64 ± 4
LV end-diastolic volume (ml)	96 ± 22	105 ± 24	97 ± 22
LV end-systolic volume (ml)	38 ± 11	44 ± 16	36 ± 12

**p < 0.01 diabetics vs controls,^†^p < 0.05 diabetic-normotensive patients vs diabetic-hypertensive patients.

The diastolic function, evaluated as the mean of E/A ratios and DT, was similar comparing diabetic normotensive to diabetic hypertensive patients but was significantly different when both the diabetic groups of patients were compared to normal subjects (E_pv_/A_pv _1.0 in both diabetic populations vs. 1.4 in controls, p < 0.01; DT 182 msec and 190 msec in diabetics respectively normotensives and hypertensives vs. 139 in controls, p < 0.01), although within the normal range (Table 3). The prevalence of abnormal diastolic filling was equal in the diabetic hypertensive patients (7 patients with impaired relaxation) and diabetic normotensive patients (6 patients with impaired relaxation).

**Table 3:** Myocardial Performance Index and Doppler time intervals.

***Parameters***	***Diabetes mellitus***	***Diabetes mellitus with hypertension***	***Controls***
LV MPI	0.49 ± 0.10*	0.49 ± 0.12**	0.39 ± 0.10
LV ICT (ms)	59 ± 22	60 ± 24	50 ± 11
LV IRT (ms)	78 ± 24^†^	80 ± 33^†^	62 ± 14
LV ET (ms)	281 ± 23	285 ± 25	295 ± 30
E-wave deceleration time (ms)	182 ± 51**	190 ± 59**	139 ± 16
E_pv _(cm/s)	67 ± 9*	71 ± 11	76 ± 16
E_tvi _(cm)	9 ± 2	9 ± 3	10 ± 2
A_pv _(cm/s)	70 ± 13**	76 ± 14**	57 ± 13
A_tvi _(cm)	6 ± 2	7 ± 2^†^	5 ± 2
E_pv_/A_pv_	1.0 ± 0.2	1.0 ± 0.2**	1.4 ± 0.3
E_tvi_/A_tvi_	1.5 ± 0.4**	1.4 ± 0.5**	2.0 ± 0.6

*p < 0.02 diabetics vs controls, **p < 0.01 diabetics vs controls, ^†^p < 0.05 diabetics vs controls.

LV, left ventricle; MPI, myocardial performance index; ICT, isovolumic relaxation time; IRT, isovolumic contraction time; ET, ejection time; E, early diastolic filling wave; A, atrial contraction wave; pv, peak velocity; tvi, time velocity integral.

**Figure 2:**
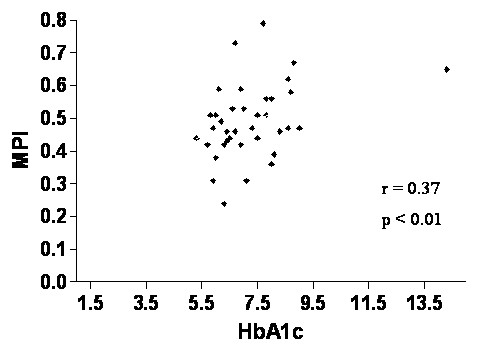
Relationship between Myocardial Performance Index and glycated haemoglobin in all diabetic patients.

MPI was significantly higher in both diabetic groups compared to controls (Table 3). This was primarily due to a significant prolongation of IRT (78 and 80 msec in diabetics normotensives and hypertensives respectively vs. 62 msec in controls, p < 0.05). The MPI was similar in the two diabetic groups independently of the diastolic dysfunction. MPI correlated to HbA1c significantly in all patients (r = 0.37, p < 0.01) (Figure 2). In particular HbA_1c _directly correlated to ICT (r = 0.29, p < 0.05) and inversely with ET (r = - 0.29, p < 0.05). No correlations were found between some clinical parameters (diabetes duration, systolic and diastolic blood pressure, patients age) and echocardiographic parameters (LV mass index, wall thickness, LV EF or FS, E/A ratios and DT.

## Discussion

In a previous study Mishra and coworkers have shown a limited role for MPI in the assessment of cardiac function in populations with multiple coronary risk factors but free of clinical cardiovascular disease [22], although some studies demonstrated an increase of MPI in type 2 diabetic patients free of coronary artery disease [9,23]. To our knowledge, our study is the first that identify the earliest cardiac performance abnormalities at echocardiography in a homogeneous group of type 2 diabetic patients with short duration of disease without coronary artery disease, independently of hypertension occurrence and diastolic function. In both diabetic groups the mean E/A ratio was lower compared to controls, although within the normal range. MPI increase was mainly due to a prolongation of IRT. Our data show that the global myocardial performance dysfunction may precede the diastolic dysfunction, therefore it represents the earliest echocardiographic sign evidence of diabetic cardiomyopathy. Several reports attempted to determine the prevalence of LV diastolic dysfunction in middle-aged asymptomatic subjects with type 2 diabetes [24,25]. However, these studies, which used Doppler assessment of transmitral flow velocity, could have underestimated the prevalence of LV diastolic dysfunction, because they neglected to account for pseudonormal patterns of ventricular filling, which are often noted in the evaluation of LV diastolic function [26]. In our study, the diastolic parameters were detected during the second phase of Valsalva manoeuvre in order to discriminate subjects with normal diastolic function from subject with "pseudonormalized" pattern.

The mechanisms behind LV performance dysfunction remain largely unknown. In normotensive and free of ischemic heart disease patients, the metabolic abnormalities may play a major role. In fact, we found a correlation between myocardial performance index and HbA_1c _(Figure 2). Nevertheless, the clinical value of this correlation, although statistically significant, will be confirmed with a prolonged follow-up and increasing the sample size. The impact of the coexistence of diabetes and hypertension on LV function has recently been investigated both in the Strong Heart study and the HyperGEN study [27-29], where apparent implications of a specific diabetes effect on LV relaxation of the myocardium were found in hypertensive type 2 diabetic patients [27]. This can be due to impaired glycemic control, microangiopathy or interstitial accumulation of elastin and collagen which also increase LV stiffness and mass in diabetic patients [30]. Ren et al demonstrated that short-term hyperglycaemia modifies the cardiomyocites contraction and relaxation in an experimental model of isolated ventricular myocites [31]. In addition, a study of Fang et al performed on diabetic patients hyperglycaemia and insulin resistance are able to induce functional and structural changes of cardiomyocites, which lead to progressive deterioration of regional and global myocardial dynamics [32]. In our study we used an echo approach for the evaluation of subclinical myocardial involvement, that represents a main advantage due to the radiation – free nature of ultrasound [33].

Our findings may have important clinical implications to the early identification of subclinical myocardial performance abnormalities with normal cardiac function at the conventional echocardiography. MPI provides an easy early phase index of diabetic cardiomyopathy that precede diastolic dysfunction, which monitors the natural history of the diabetic disease. Furthermore, MPI is useful for the indirect assessment of the metabolic control or for suggesting an early pharmacological treatment. Finally, MPI detects whether such abnormalities can modify or revert as a response to an optimal metabolic control and/or pharmacological treatment. The early diagnosis of a pre-clinical diabetic cardiomyopathy with MPI is useful for the appropriate clinical testing of new therapeutic approaches in the diabetic population.

A study limitation is represented by the patients sample, which is limited in size. In addition, the diabetes therapy was different in the two groups of patients although according to the guidelines. Moreover, we know that the exercise ECG in diabetic patients has a low sensitivity and subclinical atherosclerosis cannot be fully excluded. However, our patients were asymptomatic, free of previous history of coronary artery disease and the diabetes was of recent onset. We believe that the exercise test in patients with these characteristics can represents a reliable test with a higher sensitivity.

Beside, in common with other investigations on the MPI, we did not evaluate the effects of abnormalities of late diastolic compliance. However, when present, these are associated with raised LV filling pressure, which would shorten IRT and thus reduce the MPI, counterbalancing the prolongation due to those of early diastole. Finally, no outcome data are available at the moment.

## Conclusion

In conclusion, subtle abnormalities of LV myocardial performance are detected by means of MPI during the early stage of type 2 diabetes, independently of the hypertension presence, and it broadens the spectrum of pre-clinical diabetic cardiomyopathy.

## Authors' contributions

PP carried out all the two-dimensional and Doppler echocardiograms and participated in the sequence alignment and drafted the manuscript, FBS participated in the sequence alignment and drafted the manuscript, EC participated in the sequence alignment, AP participated in the sequence alignment, RI participated in the sequence alignment, MM selected the diabetic patients. MG participated in the design of the study and performed the statistical analysis, AB participated in the design of the study and performed the statistical analysis, MDC participated in the design of the study and drafted the manuscript, VM selected the patients. All authors read and approved the final manuscript.
